# Increasing the Participation: Another Factor

**DOI:** 10.1371/journal.pmed.0030250

**Published:** 2006-05-30

**Authors:** Ahmad A Sabri, Mohammad Ahad Qayyum

**Affiliations:** **1**Punjab Medical CollegeFaisalabadPakistan

The Perspective by Aziz Sheikh [ 1] has validly highlighted the lack of involvement of ethnic minorities in research studies, focusing on identifying the factors responsible for it. Apart from possible factors already discussed, another important factor that needs to be taken into consideration is the number of illegal immigrants in the United States and the United Kingdom. There are approximately 7 million illegal immigrants in the US (about 10% of 69.96 million ethnic minorities), and the number is growing by half a million each year [ 2, 3]. The UK also shares the same trend, with 500,000 illegal immigrants (about 10.86% of 4.6 million ethnic minorities) [ 4, 5].

In this regard, the apprehension of being traced as illegal immigrants poses a definite barrier to participating in any research activity. It is indeed a sensitive issue. Similar to the doctor–patient relationship, a confidential channel should be ensured between the researcher and the participant. It is the duty of the researcher to explain during the time of consent that this research has nothing to do with participants' citizenship status, and no information will be used in any way against them. Legislation must be passed to provide legal immunity to all participants regardless of their legal status. I believe adopting such methodology will increase the number of participants belonging to ethnic minorities. Moreover, research would be carried out on a truly representative structure of the local population.
